# Alterations of Gut Microbiota and Blood Lipidome in Gestational Diabetes Mellitus With Hyperlipidemia

**DOI:** 10.3389/fphys.2019.01015

**Published:** 2019-08-06

**Authors:** He Liu, Li-Long Pan, Siting Lv, Qin Yang, Hao Zhang, Wei Chen, Zhuwu Lv, Jia Sun

**Affiliations:** ^1^State Key Laboratory of Food Science and Technology, Jiangnan University, Wuxi, China; ^2^School of Food Science and Technology, Jiangnan University, Wuxi, China; ^3^School of Medicine, Jiangnan University, Wuxi, China; ^4^Department of Obstetrics, Wuxi People’s Hospital Affiliated to Nanjing Medical University, Wuxi, China

**Keywords:** gestational diabetes mellitus, hyperlipidemia, gut microbiota, plasma lipidome, omics

## Abstract

Clinical gestational diabetes mellitus (GDM) is frequently associated with hyperlipidemia comorbidity. Altered human gut microbiome has been linked to GDM and hyperlipidemia, respectively but not the comorbid condition. We hypothesize that the occurrence of hyperlipidemia with GDM may be characterized by distinguishable gut microbiome and blood metabolomic patterns. We presented comprehensive microbiomic coupled with lipidomics analyses to characterize gut microbiota and lipometabolism of plasma samples in women with GDM only, hyperlipidemia only and those with diabetes plus hyperlipidemia, and to explore association of the gut microbiota composition with blood lipid profiles and clinical parameters of gestational diabetes with or without commodity. We found that the relative abundance of bacterial taxa *Streptococcus*, *Faecalibacterium*, *Veillonella*, *Prevotella*, *Haemophilus* and *Actinomyces* was significantly higher in diabetes plus hyperlipidemia cohorts. Moreover, several bacteria were correlated with fasting plasma glucose and blood lipid levels of the participants with GDM and hyperlipidemia. The altered plasma lipidome in subjects with diabetes plus hyperlipidemia suggested that characteristic blood lipid profiles were associated with the pathogenesis of gestational diabetes plus hyperlipidemia. Collectively, this study provides insights on changes in fecal microbiota and plasma lipidome to predict and characterize the development of gestational diabetes with lipid metabolic abnormality.

## Introduction

The prevalence of gestational diabetes mellitus (GDM) is rapidly increasing worldwide, constituting an important health problem and a major challenge for obstetrics practice (Ferrara, 2007). Changes in the lipid metabolism are related to estrogen stimulation and insulin resistance. Hyperlipidemia is a common comorbidity in pregnancy. In women with GDM, the physiological change of lipids is amplified and may indicate underlying metabolic disturbance during pregnancy (Carpenter, 2007).

Gut dysbiosis plays a vital role in abnormal host metabolism, as recently demonstrated in studies of type 2 diabetes (T2D) and obesity (Karlsson et al., 2013). *Prevotella* and *Bacteroides* have been identified as the main species contributing to insulin resistance and glucose intolerance (Pedersen et al., 2016). While the impact of gut microbiota on host metabolism and metabolic diseases is well-documented (Moller, 2001; Ecker et al., 2010), only recently, studies have focused on microbiota changes to influence metabolic mechanisms during pregnancy (Koren et al., 2012). *Parabacteroides* are significantly more abundant in GDM women than in healthy pregnant women (Kuang et al., 2017). Novel relationship between gut microbiome composition and the metabolic hormonal environment in overweight and obese pregnant women at the first trimester has also been described (Gomezarango et al., 2016). These studies suggest that major shifts in the gut microbiome during pregnancy may play a crucial part in the development of GDM.

Lipid homeostasis plays a crucial role in T2D (Moller, 2001). The microbiota of T2D patients was negatively correlated with butyrate biosynthesis (Junjie et al., 2012). Studies based on 16S rRNA gene amplicon sequencing have revealed a decline in butyrate-producing bacteria and an increase of lactic acid-producing bacteria from the first to the third trimesters of pregnancy (Koren et al., 2012). Although recent studies have demonstrated changed gut microbiota composition in individuals with T2D and those with comorbid conditions such as hyperlipidemia (Suez et al., 2016), the relationship between the gut microbiota and plasma lipidome in GDM with hyperlipidemia remains to be clarified.

Here we hypothesized that GDM with hyperlipidemia comorbidity is associated with distinct gut microbiota profile and plasma lipidome. To this end, in the current study, we investigated the changes in gut microbiota profiles compositions and plasma lipidome of women with GDM only, hyperlipidemia and those with GDM plus hyperlipidemia, and the correlations between the profiles and clinical biochemical parameters of patients.

## Materials and Methods

### Recruitment of Subjects and Sample Collection

During September 2017 to March 2018, we selected pregnant women who were referred to a 75 g oral glucose tolerance (OGTT) in their third trimester (27–33 weeks) at Wuxi People’s Hospital. 45 individuals were divided into four cohorts according to fasting plasma glucose level (FPG), 2-h postprandial glucose level (2 h PG), triglyceride level (TG) and total cholesterol (TC): 11 were allocated to the control, 11 to the GDM only (HG) cohort (based on GDM diagnosis standard, FPG ≥ 5.1 mmol/L or 1 h PG ≥ 10.0 mmol/L or 2 h PG ≥ 8.5 mmol/L), 11 to the hyperlipidemia only (HF) cohort (TG ≥ 1.65 mmol/L and TC ≥ 5.98 mmol/L), 12 to the GDM plus hyperlipidemia (M) cohort. The participants had been previously diagnosed as healthy, with GDM only or with GDM plus hyperlipidemia by clinicians in local provincial or municipal hospitals in China. Written informed consent was obtained from the participants prior to enrollment, and approval for the study was obtained from the Ethics Committee of the Wuxi People’s Hospital (reference number: KYLLH2018032). Women with antibiotic use in the previous 3 months or active smoking were excluded. Most recent FPG, 2 h PG, TG, TC, high-density lipoprotein (HDL), high-density lipoprotein (LDL) values of each participants were obtained from their medical records. Serum aliquots were stored at −80 C. Refrigerated fecal samples were collected by nurses and stored within 1 day after collection at −80°C until DNA extraction.

### DNA Extraction, PCR and HiSeq Sequence

Total bacterial genomic DNA samples were extracted from all samples using the PowerMax (stool/soil) DNA isolation kit (MoBio Laboratories, Carlsbad, CA, United States), following the manufacturer’s instructions, and stored at −20°C prior until further analysis. The quantity and quality of extracted DNAs were measured using the NanoDrop ND-1000 spectrophotometer (Thermo Fisher Scientific, Waltham, MA, United States).

### 16S rRNA Amplicon Pyrosequencing

PCR amplification of the bacterial 16S rRNA gene V3-V4 regions was performed using the forward primer 515F (5′–GTGC CAGCMGCCGCGGTAA–3′) and the reverse primer 806R (5′–GGACTACHVGGGTWTCTAAT–3′). Sample-specific 7-bp barcodes were incorporated into the primers for multiplex sequencing. The PCR components contained 25 μl of Phusion High-Fidelity PCR Master Mix, 3 μl (10 μM) of each Forward and Reverse primer, 10 μl of DNA Template, and 6 μl of ddH_2_O. Reactions were conducted under the following conditions: initial denaturation (98°C, 30 s), 25 cycles of 98°C (15 s), annealing at 58°C (15 s), extension at 72°C (15 s) and a final extension at 72°C (1 min). PCR amplicons were purified with Agencourt AMPure XP Beads (Beckman Coulter, Indianapolis, IN, United States) and quantified using the Pico Green dsDNA Assay Kit (Invitrogen, Carlsbad, CA, United States). After the individual quantification step, amplicons were pooled in equal amounts, and pair-end 2 × 150 bp sequencing was performed using the Illumina HiSeq4000 platform at GUHE Info technology, Co., Ltd (Hangzhou, China).

### Sequence Analysis

The Quantitative Insights Into Microbial Ecology (QIIME, v1.9.0) pipeline was employed to process the sequencing data, as previously described (Caporaso et al., 2010). Briefly, raw sequencing reads with exact matches to the barcodes were assigned to respective samples and identified as valid sequences. The low-quality sequences were filtered through the following criteria: sequences that had a length of <150 bp, sequences that had average Phred scores of <20, sequences that contained ambiguous bases, and sequences that contained mononucleotide repeats of >8 bp. Paired-end reads were assembled using FLASH. Operational taxonomic unit (OTU) picking using Vsearch v1.11.1, included dereplication (–derep_fulllength), cluster (–cluster_fast,–id 0.97), detectection of chimeras (–uchime_ref). A representative sequence was selected from each OTU using default parameters. OTU taxonomic classification was conducted by VSEARCH searching the representative sequences set against the SILVA128 database.

To exclude the negative control samples, a blank control (pure water sample) and a positive control (*E*. *coli* DNA alone) were always included for DNA extraction and PCR procedures on 96-well plates. If the blank control had a band in the PCR process suggesting undesired contamination, the samples would be re-extracted and PCR re-performed. If there was no band in the blank control, and the *E. coli* positive control showed the right band, sequencing could be performed. After sequencing, if the blank control sample had more than 1000 reads of sequencing data (100,000 normal samples), contamination assessment would be performed using^1^. If the *E. coli* sample was sequenced and the OTU ratio of the non-Enterobacteriaceae sequence exceeded 3%, it indicated that the whole samples was contaminated, and we would re-sequence the samples.

To minimize the difference of sequencing depth across samples, an averaged, rounded rarefied OTU table was generated by averaging 100 evenly resampled OTU subsets under the 90% of the minimum sequencing depth for further analysis.

### Lipid Extraction

Methanol (0.3 ml) was added to a 40 μl blood sample aliquot, which was placed into a 1.5 ml tube, and the tube was vortexed. Then, 1 ml of MTBE was added and the mixture was incubated for 1 h at room temperature in a shaker. Phase separation was induced by adding 0.25 ml of MS-grade water. Upon 10 min of incubation at room temperature, the sample was centrifuged at 1,000 g for 10 min. The upper (organic) phase was collected and dried in a vacuum centrifuge. Extracted lipids were dissolved in 200 μl of CHCl3/methanol/water (60:30:4.5, v/v/v) for storage (Matyash et al., 2008).

### Lipid Analysis by Gas Chromatography and Mass Spectrometry

The lipids were analyzed by LC-MS/MS using a Thermo Vanquish HPLC (Thermo Fisher Scientific, Germering, Germany). MS was performed with heated ESI source in positive and negative mode, respectively. The spray voltage was set to 3.5 kV for positive and −2.8 kV for negative mode, and ion transfer capillary was 325°C. Nitrogen was used as both sheath gas and auxiliary gas and was set to 35 and 15 arbitrary units, respectively, and the auxiliary gas temperature was 250°C. For MS/MS, higher-energy collision dissociation (HCD) with nitrogen gas and step collision energy (NCE) of 20, 30, 50 for positive mode, and 20, 30, and 50 for negative mode were used to present a broader range of fragment ions and collected as much informative data as possible. MS data were acquired in the scan range of m/z 80–1200 and were processed using Xcalibur software version 4.0 (Thermo Scientific, San Jose, CA, United States).

### Lipidomics Data Processing

LipidSearch software (Thermo Fisher Scientific, San Jose, CA, United States) was used to identify lipid molecular species and extractability evaluation was assessed by comparing peak abundances. Parent and product search mode for ceramides (CER), cholesteryl esters (Che), diacylglycerols (DG), dimethyl-phosphatidylethanolamine (dMePE), fatty acids (FA), lysophos-phatidylcholines (LPC), lysophosphatidylethanol (LPEt), lyso-phosphatidylglycerol (LPG), lysophosphatidylinositol (LPI), monoglyceride (MG), (o-acy)-1-hydroxy fatty acid (OAHFA), platelet-activating factor (PAF), phosphatidylcholines (PC), phos-phatidylethanolamines (PE), phosphatidylethanol (PEt), phos-phatidylglycerols (PG), sphingomyelin (phSM), phosphatidyl-inositol (PI), phosphatidylinositol (PIP), phosphatidylinositol 3 (PIP_3_), sphingomyelins (SM), sphingoshine (So), and triacylglycerols (TG) were used in the current study. Parent search mode was identified based on accurate mass of precursor ions, and product search mode was identified based on the accurate mass of precursor ions and MS/MS spectral pattern.

### Bioinformatics and Statistical Analysis

Gut microbiota sequence data analyses were mainly performed using QIIME and R packages (v3.2.0). OTU-level alpha diversity indices, such as Chao1 richness estimator, ACE metric (Abundance-based Coverage Estimator), PD_whole_tree, Shannon diversity index, and Simpson index, were calculated using the OTU table in QIIME. Beta diversity analysis was performed to investigate the structural variation of microbial communities across samples using UniFrac distance metrics and visualized via non-metric multidimensional scaling (NMDS). Significant differences in the UniFrac distances for pairwise comparisons among four groups were determined using Student’s *t*-test and the Monte Carlo permutation test with 1000 permutations, and visualized through the box-and-whiskers plots. The significance of differentiation of microbiota structure among groups was assessed by PERMANOVA (Permutational multivariate analysis of variance) using R package “vegan”. The taxonomy compositions and abundances were visualized using MEGAN (Huson et al., 2011) and GraPhlAn (Zaura et al., 2009). Venn diagram was generated to visualize the shared and unique OTUs among samples or groups using R package “Venn Diagram”, based on the occurrence of OTUs across samples/groups regardless of their relative abundance (Zaura et al., 2009). Taxa abundance at the phylum, class, order, family, genus and species levels was statistically compared among samples or groups by Kruskal test from R stats package.

Based on the relative intensities of the lipids from the normalized profiling data, One-Way Analysis of Variance (ANOVA) followed by *Post Hoc* Tests were used to reveal the significant differences of the lipids among each group. Results were considered statistically significant when *P* < 0.05.

## Results

### Cohort Description

The baseline characteristics of the participants in each cohort are displayed in Table 1. The mean age of the pregnant women showed no difference among NC, HG, HF and M cohorts. The women diagnosed with GDM had higher plasma glucose (0 h or 2 h, *P* < 0.001) than the control cohort and HF cohort during OGTT. The group of women with hyperlipidemia only or plus GDM showed significantly higher levels of TC and TG (*P* < 0.001) than control cohort and HG cohort. Levels of HDL (*P* < 0.05) and LDL (*P* < 0.001) were significantly different between HF cohort, M cohort and control cohort, HG cohort.

**TABLE 1:** Characteristics of the NC, HG, HF, and M cohorts^a^.

**Characteristics**	**NC**	**HG**	**HF**	**M**	***P*-value**
Age	28.2 ± 0.0.8	29.3 ± 0.9	27.3 ± 0.6	29.1 ± 0.7	0.2
Gestational week at examination	32.7 ± 0.3	31.2 ± 0.5	32.2 ± 0.8	31.8 ± 0.8	0.4
BMI (kg/m^2^)	26.7.0 ± 0.6	26.6 ± 1.1	26.9 ± 0.7	25.5 ± 0.8	0.6
Ethnicity,%					
Xanthoderm	11	11	11	12	
Other	0	0	0	0	
FPG (mmol/L)	4.5 ± 0.08^∗‡^	5.0 ± 0.09^∗§^	4.6 ± 0.1^§¶^	5.1 ± 0.2^‡¶^	0.0006
2-h PG (mmol/L)	6.6 ± 0.37^∗‡^	8.3 ± 0.4^∗¥^	6.1 ± 0.3^¥¶^	8.1 ± 0.4^‡¶^	<0.0001
TC (mmol/L)	5.1 ± 0.2^†‡^	5.6 ± 0.1^§¶^	6.6 ± 0.3^†§^	7.2 ± 0.2^‡¶^	<0.0001
TG (mmol/L)	2.1 ± 0.08^†‡^	2.4 ± 0.08^¶^	2.7 ± 0.1^†^	3.3 ± 0.3^‡¶^	<0.0001
HDL (mmol/L)	1.8 ± 0.08^‡^	1.9 ± 0.05^¶^	1.7 ± 0.1	1.5 ± 0.07^‡¶^	0.02
LDL (mmol/L)	2.1 ± 0.2^†‡^	2.4 ± 0.1^§¶^	3.6 ± 0.2^†§^	3.5 ± 0.2^‡¶^	<0.0001

*Data are mean ± SEM, unless otherwise stated. Clinical characteristics and biochemical variables of four cohorts at 24–28 weeks’ gestation. ^a^NC, control; HG, gestational diabetes; HF, hyperlipidemia; M, gestational diabetes mellitus with hyperlipidemia. ^b^Independent ANOVA analysis was used to test for significant differences (P < 0.05) in each variable between the four cohorts. ^∗^, ^†^, ^‡^, ^§^, and ^¶^ indicate significant differences between the NC and HG cohorts, between the NC and HF cohorts, between the NC and M cohorts, between HG and HF cohorts, and between HG and M cohorts, respectively.*

### Sequencing Data

To analyze the gut bacterial profiles, the overall microbiota from control non-diabetic pregnant women (NC), women with GDM, women with hyperlipidemia and women with GDM plus hyperlipidemia were sequenced. The mean read length was 262 bp (ranging from 209 to 290).

As shown in Supplementary Table S1, the number of tags and OTUs was significantly higher in the healthy cohort compared to those in HG, HF and M cohorts (Supplementary Figure S1). The diversity of the fecal microbiota was determined by the α-diversity analysis. It showed significantly lower diversity for HG, HF and M cohorts than that for the control group. The α-diversity differences were consistent across different metrics between the control group and HG, HF and M cohorts, i.e., chao1, Shannon index, phylogenetic diversity (PD) whole tree, and observed species (Supplementary Figure S2).

### Comorbid Conditions With GDM Were Associated With Alterations in the Gut Microbiota Profiles

Further Spearman correlation analysis was performed to detect whether and how the gut microbiota could be attributed to blood glucose and serum lipid levels. Strong correlations (*P* < 0.01) were found between FPG and α-diversity indices (Shannon index, Chao1 index and Ace index) (Table 2). The Spearman correlation coefficients revealed several significant linkages, including a negative association between FPG (*P* < 0.01), TG (*P* < 0.01) and TC (*P* < 0.05) and the Shannon index; a negative association between FPG (*P* < 0.01), TG (*P* < 0.01), TC (*P* < 0.001) and LDL (*P* < 0.01) and the Chao1 index; a negative association between FPG (*P* < 0.01), TG (*P* < 0.01), TC (*P* < 0.001) and LDL (*P* < 0.01) and the ace index (Table 2).

**TABLE 2:** α diversity indices and their correlation with clinical characteristics and biochemical variables in in the NC, HG, HF, and M cohorts.

**α diversity**	**FPG**	**2hPG**	**TG**	**TC**	**HDL**	**LDL**
Shannon	−0.3968^∗∗^	0.2441	−0.4205^∗∗^	−0.3059^∗^	−0.01552	−0.2603
chao1	−0.409^∗∗^	−0.2366	−0.4081^∗∗^	−0.5089^∗∗∗^	0.2382	−0.41^∗∗^
ace	−0.407^∗∗^	−0.2515	−0.4011^∗∗^	−0.5146^∗∗∗^	0.2422	−0.4151^∗∗^

*Data are Spearman correlation coefficients. Significant correlations shown in boldface. ^∗^P < 0.05; ^∗∗^P < 0.01; ^∗∗∗^P < 0.001.*

**FIGURE 1:**
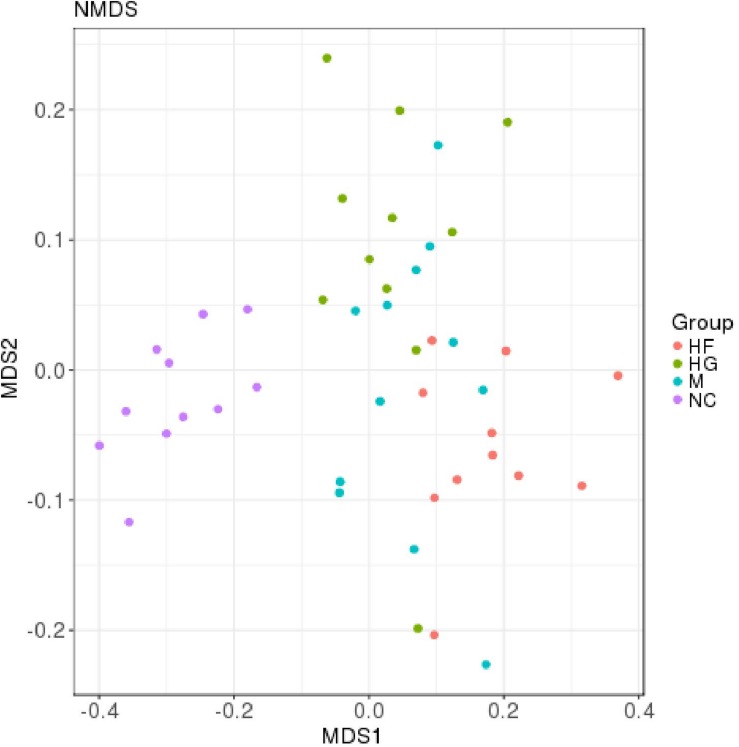
Distribution of gut microbiota according to different gestational status, related to Supplementary Figure S1. Microbial communities clustered using NMDS analysis. Each point represents one sample of NC (purple, *n* = 11), HG (green, *n* = 11), HF (red, *n* = 11) and M (blue, *n* = 12) pregnant women. The distance among different samples reflects the comparability of four cohorts.

**FIGURE 2:**
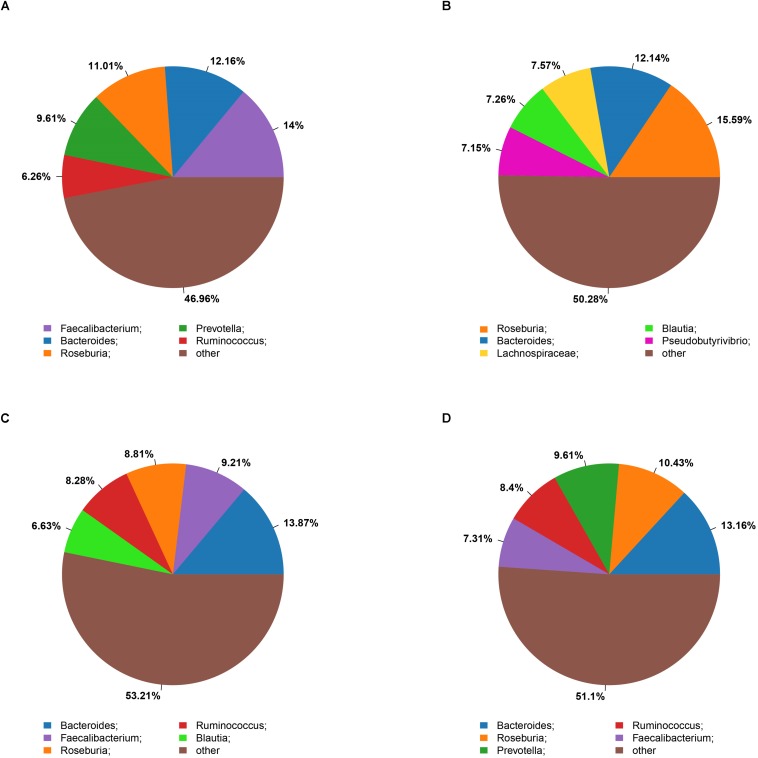
Summary of bacterial genera detected in the four cohorts **(A–D)** indicate the top five most abundant genera detected in the NC **(A)**, HG **(B)**, HF **(C)**, and M **(D)** cohorts, respectively.

The microbiota analysis with NMDS identified differentially-abundant fecal bacterial taxa of the four cohorts (Figure 1). Our results showed that the diversity of fecal microbiota was mainly related to 6 phyla: *Firmicutes*, *Proteobacteria*, *Bacteroidetes*, *Actinobacteria*, *Verrucomicrobia* and *Tenericutes* (Supplementary Figure S3). The predominant genera in the NC and HF cohorts were *Faecalibacterium*, *Bacteroides* and *Roseburia;* in the HG cohorts were *Roseburia, Bacteroides and Lachnospiraceae;* in M cohorts were *Bacteroides*, *Roseburia* and *Prevotella* (Figure 2).

**FIGURE 3:**
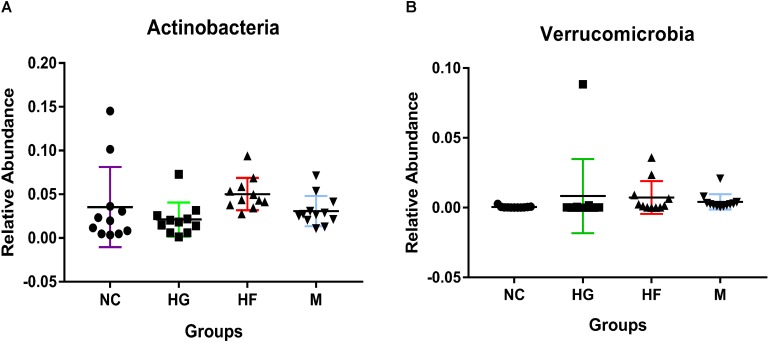
Phylum-level relative abundance of bacteria that in fecal samples was significantly different between the four cohorts NC, HG, HF, and  M  cohorts. Kruskal–Wallis test was used to compare the differences in the relative abundance of bacterial phylum between the four cohorts. **(A,B)** Indicate the significant differences (*P* < 0.05) between the four cohorts.

The Kruskal test revealed the relative abundance of two dominant fecal phyla differed significantly among the four groups, with a higher relative abundance of *Actinobacteria* in the HF group and *Verrucomicrobia* in the HG group (Figure 3 and Supplementary Table S2). Relative abundance at class, order and family levels was similar among the four groups. At the genus level, NC cohorts had a significantly higher abundance of *Faecalibacterium*, while M cohorts were significantly enriched for *Streptococcus*, *Veillonella*, *Prevotella*, *Haemophilus* and *Actinomyces* (Supplementary Table S3).

The relationship between the fecal microbiome composition and individual characteristics such as FPG, 2 h PG, TG, TC, HDL, and LDL was also assessed. At phylum, both TC and TG were positively correlated with the relative abundance of *Proteobacteria* (*P* < 0.05). 2 h PG was negatively correlated with the relative abundance of *Actinobacteria* (*P* < 0.05) (Supplementary Table S4). There were no significant associations of FPG, TG, and HDL with the gut microbiota. At genus, by the Kruskal test, we identified that lower relative abundance of *Faecalibacterium* was associated with increased TG (*P* < 0.01). In addition, increased relative abundance of *Streptococcus* and *Actinomyces* genus was positively associated with higher TC levels (P < 0.01). The genus *Veillonella* (*P* < 0.01) and *Haemophilus* (*P* < 0.05), which were positively correlated to TC levels, were negatively correlated with HDL levels (*P* < 0.05) (Table 3).

**TABLE 3:** Gut microbiota abundance (genus) and their correlation with clinical characteristics and biochemical variables in in the NC, HG, HF, and M cohorts.

	**FPG**	**2hPG**	**TG**	**TC**	**HDL**	**LDL**
*Faecalibacterium*	−0.2132	−0.2190	−0.4022^∗∗^	−0.1568	−0.03149	−0.2607
*Streptococcus*	0.0667	0.1028	0.2035	0.3875^∗∗^	−0.2192	0.2644
*Veillonella*	0.1285	0.1494	0.1062	0.3862^∗∗^	−0.3353^∗^	0.1829
*Haemophilus*	0.1568	0.2083	0.1382	0.3631^∗^	−0.3271^∗^	0.1957
*Actinomyces*	0.2293	0.1945	0.2176	0.3510^∗^	−0.2472	0.2499

*Data are Spearman correlation coefficients. Significant correlations shown in boldface. ^∗^P < 0.05; ^∗∗^P < 0.01.*

### Abnormal Lipid Metabolism in GDM and Association of Lipid Classes With Gut Microbiota

In the present study, whole plasma lipidomics was used to determine the differences between M cohort and the other three groups. Numerous lipid classes were identified by LipidSearch, including CER, Che, cPA, DG, dMePE, FA, LPA, LPC, LPG, LPI, PE, PI, PS, PG, PA, SM, So, TG, and more than 3000 lipid species were detected and identified.

**FIGURE 4:**
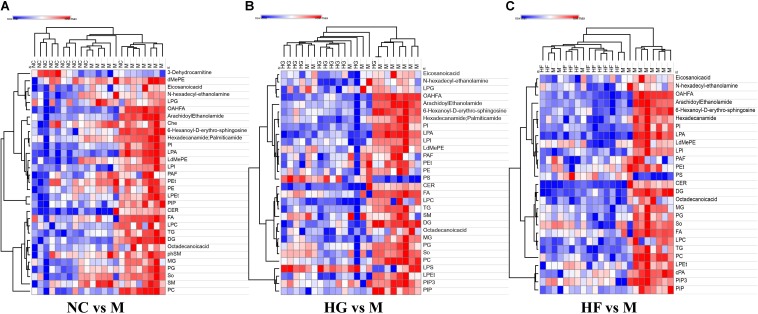
Clusters of M cohort lipid classes revealed by abundance covariation. A heatmap illustrating that the 31 lipid classes clearly segregate NC cohort and M cohort **(A)**, 27 lipid classes clearly segregate HG cohort and M cohort **(B)**, 30 lipid classes clearly segregate HF cohort and M cohort **(C)**. Each colored cell on the map corresponds to a relative concentration value in the data table, with samples in columns and features/compounds in rows. The heatmap was used to identify samples/features that are unusually high/low. The metabolite annotation is listed in Supplementary Table S5. Red lines represent metabolites that are increased and blue lines represent metabolites that are reduced relative to M cohort.

To further evaluate the difference among these four cohorts, peak areas of lipid classes were compared. Comparison of compositions of lipid classes in cohorts in positive and negative ion mode was presented in Figure 4 (*P* < 0.01) (Supplementary Table S5). This lipidomics study indicates the abnormal lipid metabolism in M cohorts. Compared with the other three groups, most lipid classes were up-regulated in M cohorts.

**FIGURE 5:**
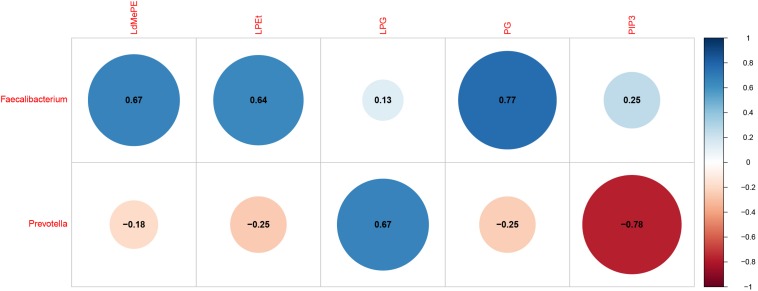
Gut microbiota (genus) associated with significantly changed lipids during pregnancy with GDM plus hyperlipidemia (M cohort). Dot Plot of correlations between bacteria genus and LdMePE, LPEt, LPG, PG, PIP_3_. The size of each point represents the correlation coefficient and the color represents positive (blue) or negative (red) relationship.

The interactions between gut microbiota and the significantly changed lipid profiles, obtained by combining datasets from both ion modes were further evaluated in M cohorts. At phylum level, the lipidomics datasets were significantly associated with *Firmicutes*, *Bacteroidetes*, *Actinobacteria* and *Tenericutes* (Supplementary Table S6). At genus level, we found that *Faecalibacterium* was positively associated with LdMePE, LPEt and PG, while *Prevotella* was positively correlated with LPG and negatively correlated with PIP_3_ (Figure 5).

## Discussion

In addition to recent report on changes in microbial compositions of pregnant women with GDM (Kuang et al., 2017; Crusell et al., 2018), our study has provided a more comprehensive analysis of the gut microbial changes of pregnant women with GDM, hyperlipidemia and GDM plus hyperlipidemia, coupled with multi-omics analysis of serum and gut samples to determine the relationship and differences of gut microbiota and lipid metabolism. By using LC-MS/MS-based untargeted lipidomics analysis, this is the first large-scale study to explore the alterations in plasma lipid patterns of individuals with GDM and hyperlipidemia.

Our results suggest that GDM, hyperlipidemia and GDM plus hyperlipidemia lower the diversity of the microbiota, as the M cohort had lower numbers of tags and OTUs compared to the HG cohorts. Among the four cohorts, subjects with hyperlipidemia had lower bacterial abundance than NC, HG, and M cohorts. It implies that hyperlipidemia has a stronger impact on gut microbiota than hyperglycemia. It has been shown that a reduced richness of gut microbiota is associated with elevated pro-inflammatory markers and insulin resistance (Le Chatelier et al., 2013).

In this study, we analyzed gut microbiota profile with highlighted differential bacteria taxa composition in relation to clinical characteristics of the four cohorts. Similarly to Luisa F and Kuang Y (Gomez-Arango et al., 2016; Kuang et al., 2017), our results identified the top four most abundant bacteria in the four cohorts were composed of non-pathogenic commensal microbiota from *Firmicutes*, *Bacteroidetes*, *Proteobacteria*, and *Actinobacteria*, with the proportion of *Actinobacteria* was higher of HF cohort. Moreover, M cohorts were associated with a higher proportion of the bacterial genus *Streptococcus*, *Faecalibacterium*, *Veillonella*, *Prevotella*, *Haemophilus* and *Actinomyces*.

As an important butyrate-producer, *Faecalibacterium prausnitzii* is known for anti-inflammatory properties (Sokol et al., 2008). It has been reported that healthy pregnant women have a lower abundance of *Faecalibacterium* in the third trimester (Koren et al., 2012). Reduced abundance of *F. prausnitzii* has been reported in non-pregnant adults with metabolic abnormalities and T2D (Zhang et al., 2013; Haro et al., 2016). Accordantly with these studies, we also found that *Faecalibacterium* was reduced in the HF, HG and M cohorts. Furthermore, we observed a negative correlation of *Faecalibacterium* with TG in pregnant women. Several earlier studies showed contrasting results in gut microbiome of GDM (Crusell et al., 2018) or urinary microbiota in T2D plus hyperlipidemia (Liu et al., 2017). It could be attributed to the different species or strains of *Faecalibacterium* involved, which could be further verified by shot-gun sequencing-based metagenomics.

Interestingly, the present microbiological assessment was that M cohort exhibited significantly higher abundance of *Actinomyces* than did HG, HF and NC cohorts, which was positively correlated with TC. *Actinomyces* are typically found in oral microbiota. Previous studies have reported that the relative abundance of *Actinomyces* was higher in individuals with a glucose diet (Matee et al., 1993) and contributed to plaque related diseases (Moore and Moore, 1994). In addition, recently, *Actinomyces* have been implicated in gastric *Actinomycosis* for causing morbid obesity after gastric bypass (Baierlein et al., 2007). Notably, we found a positive relationship between *Actinomyces*, *Streptococcus*, *Haemophilus*, *Veillonella* and TC. These bacteria could be potential targets for intervention for patients with diabetes and hyperlipidemia.

There was a significantly higher relative abundance of *Prevotella* in M cohort compared to that in the other three cohorts. Previous studies have reported that the relative abundance of *Prevotella* was positively associated with T2D and obesity (Zhang et al., 2009; Fugmann et al., 2015). Pedersen et al. identified *Prevotella* as one of the main species driving the association between biosynthesis of branched-chain amino acids and insulin resistance (Pedersen et al., 2016). As mucin degrading bacteria, *Prevotella* may contribute to increased gut permeability (Brown et al., 2011). Similar to what happens in new-onset rheumatoid arthritis (Scher et al., 2013) and obesity (Furet et al., 2010), *Prevotella* should have a strong modulatory influence on the immune system and be related to low-grade inflammation. Therefore, the high relative abundance of *Prevotella* may be a causal factor in the development of hyperglycemia plus hyperlipidemia during pregnancy.

Analysis on the relative abundance of bacteria across the four cohorts led us to a surprising discovery of *Akkermansia* genus, which belongs to the *Verrucomicrobia* phylum, both being higher in HG cohort. *Akkermansia* is known to be positively associated with better metabolic health and insulin sensitivity and inversely correlated to FPG (Hansen et al., 2012; Dao et al., 2016). In rodents, probiotic supplementation with *Akkermansia* improved glucose tolerance and insulin sensitivity (Zhao et al., 2017). Our results suggest that *Akkermansia* might have additional impact on host metabolism in pregnant women with GDM than previously described or that some unknown subspecies of *Akkermansia* play a role here, which could be further analyzed by whole-genome sequencing at a deeper taxonomic resolution.

Indeed, abnormal lipid metabolism readily occurs in diabetes. In this study, the abnormal lipid metabolism was characterized by a significant increase in GDM with hyperlipidemia, such as LPCs, SM and Cer. Plasma levels of the LPC classes have been demonstrated to be correlated with plasma level of LDL (Goncalves et al., 2012). Sphingomyelin (SM) was up-regulated in M cohort. Sphingolipid metabolism may be influenced by glucocorticoids, which can increase membrane sphingomyelin (Cohen et al., 1986). Indeed, glucocorticoids levels are found to be increased in diabetes patients. These earlier data are in agreement with our observation of increased SM with the development of GDM. The role of ceramides in insulin resistance has been intensively investigated. It has been reported that exogenous ceramide administration induces apoptosis of the islet cells in the absence of fatty acids (Shimabukuro et al., 1998). Ceramides play a vital role in TLR4-dependent insulin resistance in obesity (Shi et al., 2006). Here, we found that the alterations of *Firmicutes* and *Bacteroidetes* were associated with serum levels of ceramides. It has been found that LPG was significantly increased in T2D patients (Lu et al., 2016). Moreover, increase or decrease in production of PIP_3_ can result in obesity and diabetic phenotypes (Manna and Jain, 2015). As expected, we found that the two lipid metabolites were significantly associated with *Prevotella*. Our data provide strong evidence that gut microbiome has a hand to play in the lipid metabolic mechanisms of GDM with hyperlipidemia. However, metagenomics could be done to identify the specific species. Our results indicate that plasma lipid classes might reflect some of abnormal changes during GDM development. Various biomarkers identified may worth further evaluation in prevention and treatment of GDM with hyperlipidemia.

Several limitations were present in our study. Firstly the cohort sizes were relatively small, partly due to the stringent inclusion criterion. Furthermore, patient samples were analyzed one time per participant, which were collected in the third trimester of pregnancy. As immune and metabolic changes occur throughout pregnancy, the maternal microbial composition could be substantially- different comparing the third trimesters to the 1st months postpartum (Koren et al., 2012; Crusell et al., 2018). Here, we were unable to clarify the causal relationship between the microbiome and the development of GDM due to the cross-sectional design. In addition, environmental factors, e.g., nutrient intake and life style may affect blood glucose, lipid and fecal microbiota composition. To circumstantiate the diversification of fecal microbiota and blood lipidome observed in the current study, a larger prospective cohort with standardized food and nutrient intake to eliminate the dietary effect could be more persuasive.

Together, this study allows a better understanding on the relationship of gut microbiota and plasma lipids associated with the metabolic conditions of pregnant women. Future studies combining metagenomics, lipidomics and proteomics should be conducted to describe detailed variations in patients with GDM and comorbid hyperlipidemia and to support precise prevention and intervention strategies for GDM with hyperlipidemia.

## Data Availability

The datasets generated for this study can be found in the NCBI, https://www.ncbi.nlm.nih.gov/sra/PRJNA511904.

## Author Contributions

HL, SL, and QY performed the experiments and analyzed the data. L-LP, HZ, and WC provided intellectual inputs, contributed to the data acquisition, and critically reviewed the manuscript. JS, L-LP, and ZL designed and interpreted the experiments. HL, JS, and L-LP wrote the manuscript.

## Conflict of Interest Statement

The authors declare that the research was conducted in the absence of any commercial or financial relationships that could be construed as a potential conflict of interest.

## Footnotes

1 https://github.com/benjjneb/decontam

## Supplementary Material

FIGURE S1 Venn diagram showing the OTU distributions among the four groups.

FIGURE S2 Alpha diversity in NC, HG, HF and M pregnant women as represented by chao1 **(A)**, Shannon diversity **(B)**, PD_whole_tree **(C)** and Observed_species **(D)**. Samples were rarefied to an equal sequencing depth of 30,000 tags.

FIGURE S3 Bacterial composition using mean relative abundances at the bacterial phylum level.

TABLE S1 Richness and diversity estimators in the NC, GDM, HF, and M cohorts^a^.

TABLE S2 Phylum-level relative abundance of bacteria in fecal samples of NC, HG, HF, and M cohorts.

TABLE S3 Genus-level relative abundance of bacteria in fecal samples of NC, HG, HF, and M cohorts.

TABLE S4 Gut microbiota abundance (phylum) and their correlation with clinical characteristics and biochemical variables in in the NC, GDM, HF, and M cohorts.

TABLE S5 *P*-value of significant plasma lipids of NC, HG, HF, and M cohorts.

TABLE S6 Gut microbiota abundance (phylum) and their correlation with lipid classes variables in the M cohort.
