# Supplementary figures and images for: Alterations of Gut Microbiota and Blood Lipidome in Gestational Diabetes Mellitus With Hyperlipidemia
Source: Front Physiol. 2019 Aug 6;10:1015. doi: 10.3389/fphys.2019.01015 (PMC6691352; doi:10.3389/fphys.2019.01015)

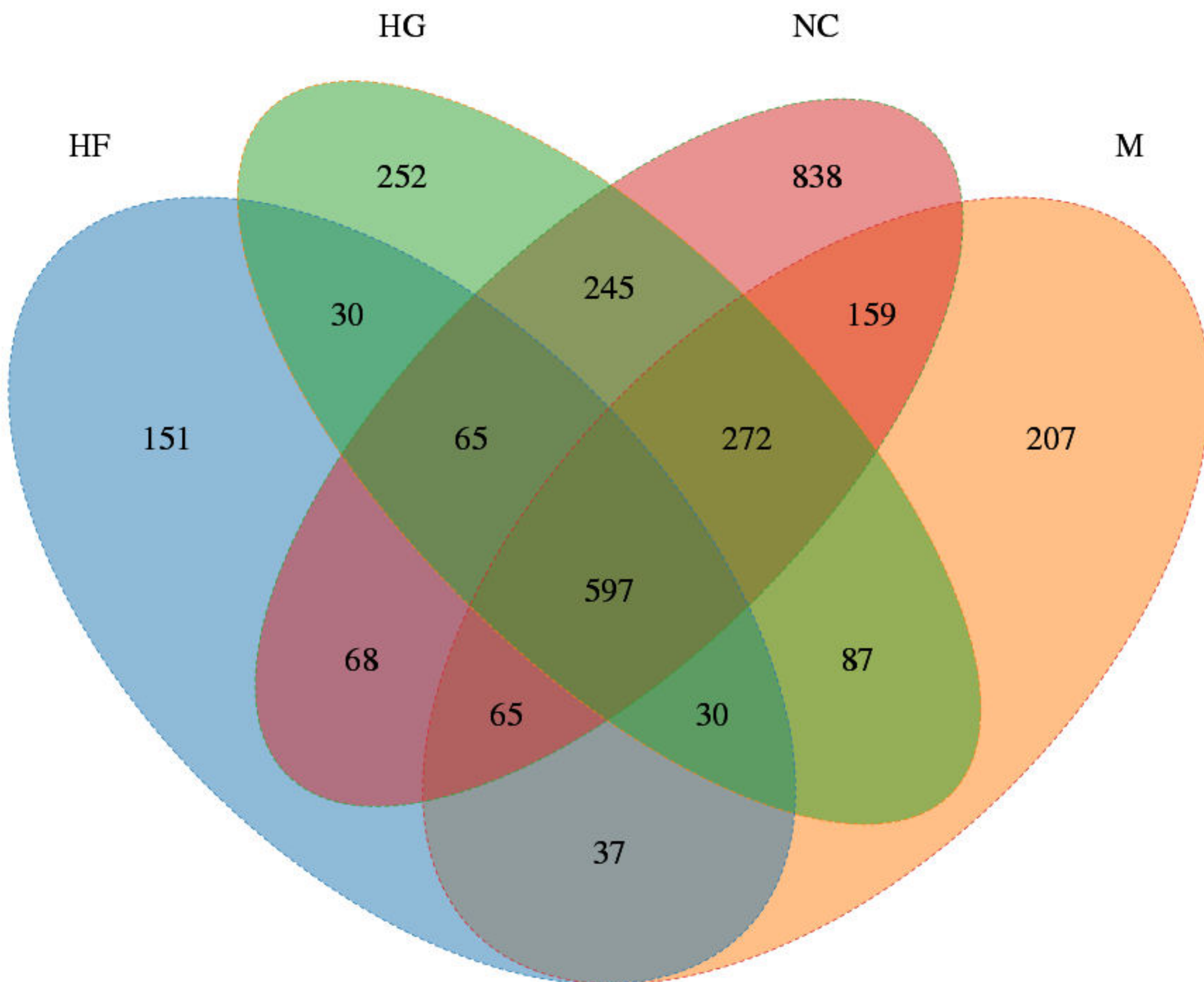

Supplement: FIGURE S1 — Venn diagram showing the OTU distributions among the four groups. [file Data_Sheet_2.PDF]

A

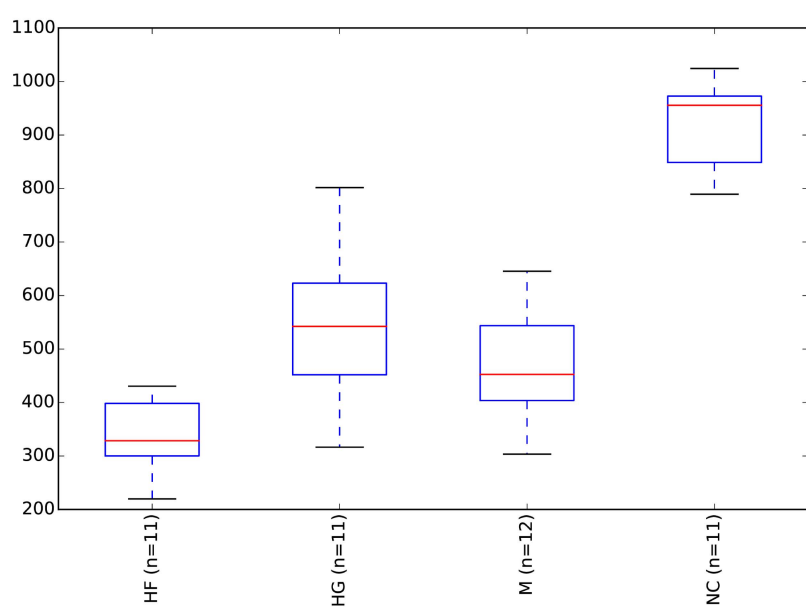

B

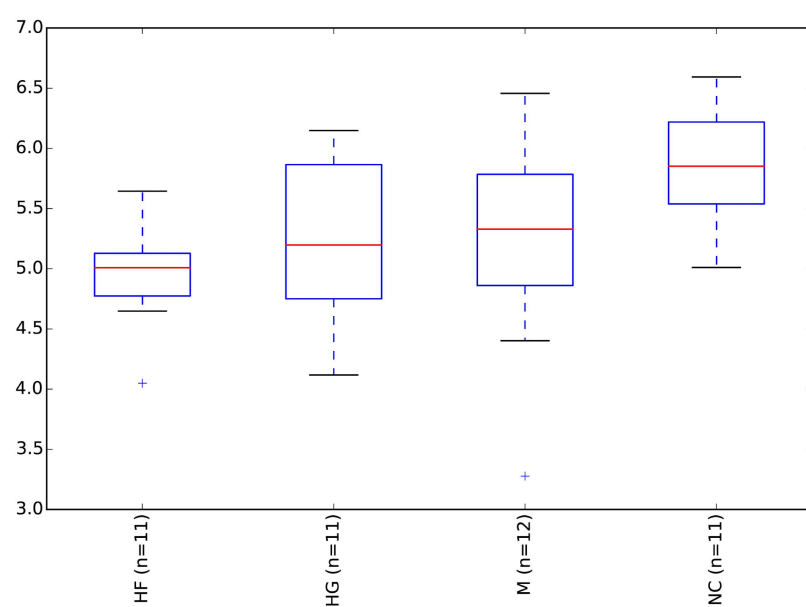

C

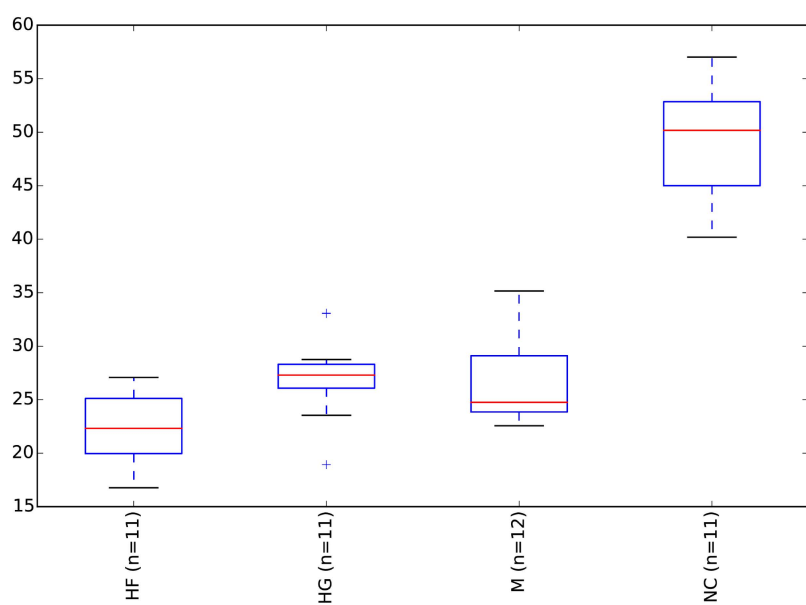

D

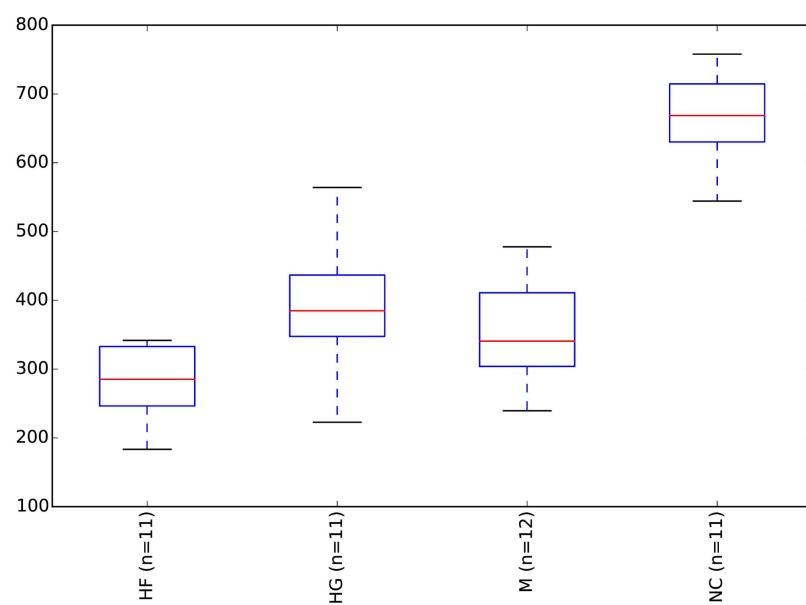

Supplement: FIGURE S2 — Alpha diversity in NC, HG, HF and M pregnant women as represented by chao1 (A), Shannon diversity (B), PD_whole_tree (C) and Observed_species (D). Samples were rarefied to an equal sequencing depth of 30,000 tags. [file Data_Sheet_3.PDF]

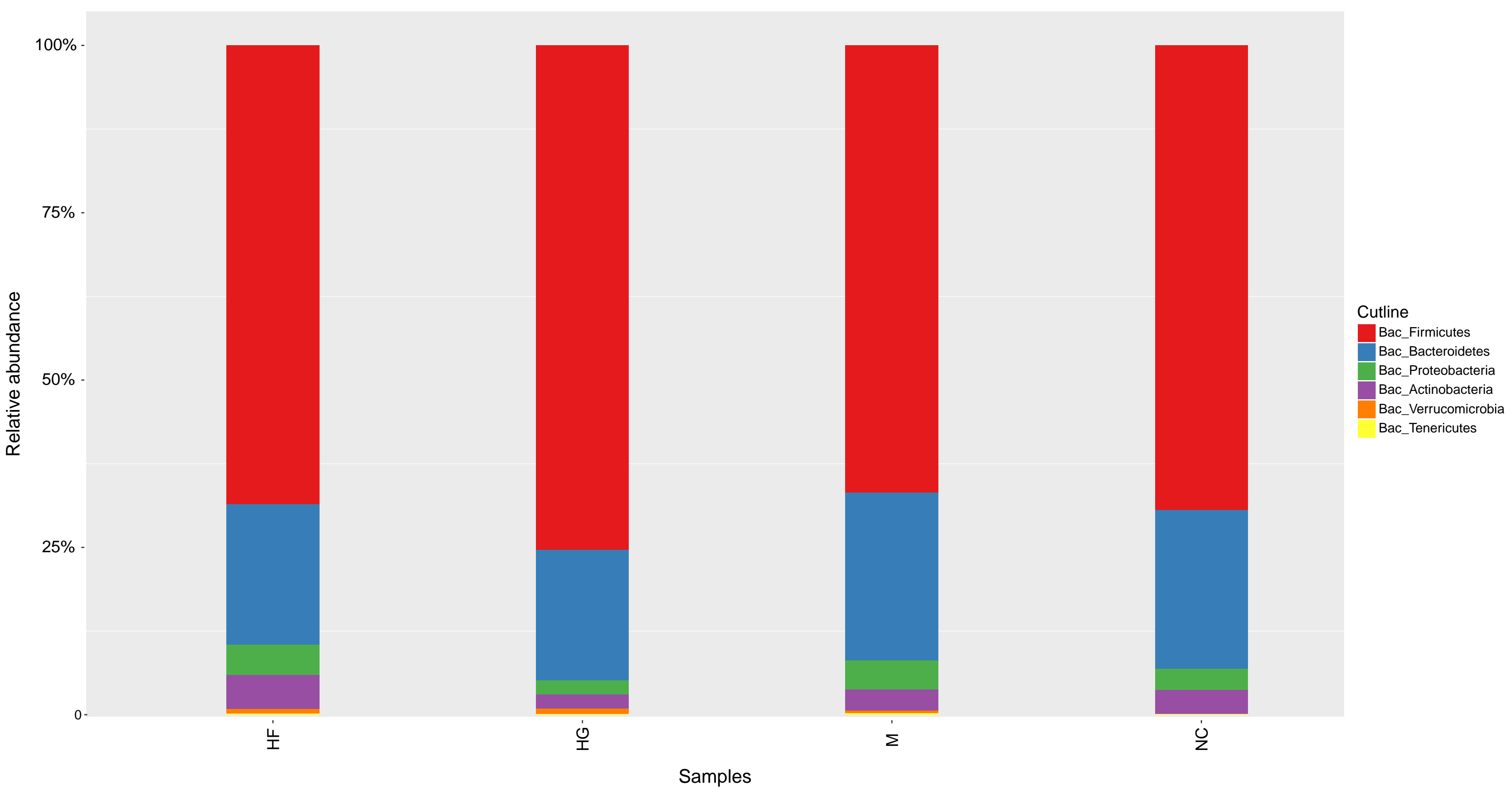

Supplement: FIGURE S3 — Bacterial composition using mean relative abundances at the bacterial phylum level. [file Data_Sheet_4.PDF]
