# Supplementary material for: Alterations of Gut Microbiota and Blood Lipidome in Gestational Diabetes Mellitus With Hyperlipidemia
Source: Front Physiol. 2019 Aug 6;10:1015. doi: 10.3389/fphys.2019.01015 (PMC6691352; doi:10.3389/fphys.2019.01015)
Supplement: TABLE S1 — Richness and diversity estimators in the NC, GDM, HF, and M cohortsa. [file Data_Sheet_1.pdf]

**Supplementary Table 1 | Richness and diversity estimators in the NC, HG, HF, and M cohorts[a].**

| Parameter | NC | HG | HF | M |
|---|---|---|---|---|
| Number of tags | 132523±2979[†‡] | 129279±3709[§] | 104564±1046[†§] | 117713±5745[‡] |
| Number of OTUs | 2309 | 1578 | 1043 | 1454 |
| Chao1 | 915.79[*,†,‡] | 536.99[*,§] | 337.69[†,§] | 464.75[‡] |
| Shannon index | 5.8395[†] | 5.2318 | 4.967[†] | 5.214 |
| Simpson index | 0.9416 | 0.9298 | 0.9259 | 0.9304 |
| PD_whole tree | 49.117[*,†,‡] | 27.182[*,§] | 22.313[†,§] | 26.96[‡] |
| Observed_species | 667.72[*,†,‡] | 395.66[*,§] | 281.31[†,§] | 354.3[‡] |

[a]HG, gestational diabetes mellitus; HF, hyperlipidemia; M, gestational diabetes mellitus plus hyperlipidemia; OTU, operational taxonomic units.

[b]The operational taxonomic units (OTUs) were defined based on a similarity threshold of 97%; Independent t-test was used to test for significant differences ($p < 0.05$) in each variable between the four cohorts. [*], [†], [‡] and [§] indicate significant differences between the NC and HG cohorts, between the NC and HF cohorts, between the NC and M cohorts, and between HG and HF cohorts, respectively. The parameters were calculated using QIIME software.

**Supplementary Table 2 | Phylum-level relative abundance of bacteria in fecal samples of NC, HG, HF and M cohorts.**

| | mean_NC | mean_HG | mean_HF | mean_M | Kruskal.test |
|---|---|---|---|---|---|
| *Actinobacteria* | 0.035322 | 0.021067 | 0.050202 | 0.03071 | 0.004082 |
| *Verrucomicrobia* | 0.000479 | 0.008284 | 0.007298 | 0.004111 | 0.001986 |

Significant differences were calculated using the Kruskal test by R. A significant difference between the groups was considered at $P < 0.05$.

**Supplementary Table 3 | Genus-level relative abundance of bacteria in fecal samples of NC, HG, HF and M cohorts.**

| | mean_NC | mean_HG | mean_HF | mean_M | Kruskal.test |
|---|---|---|---|---|---|
| *Streptococcus* | 0.002419 | 0.001865 | 0.003089 | 0.009571 | 0.000576 |
| *Faecalibacterium* | 0.136772 | 0.067333 | 0.089484 | 0.069898 | 0.009032 |
| *Veillonella* | 0.001268 | 0.000564 | 0.001833 | 0.008154 | 0.000439 |
| *Prevotella* | 7.71E-05 | 0.0029 | 0.001412 | 0.003131 | 0.001562 |
| *Haemophilus* | 0.000269 | 0.000496 | 0.000778 | 0.005828 | 0.000394 |
| *Actinomyces* | 6.82E-05 | 0.000233 | 0.000327 | 0.003236 | 1.36E-05 |
| *Akkermansia* | 0.000277 | 0.008265 | 0.006419 | 0.003619 | 0.000635 |

Significant differences were calculated using the Kruskal test by R. A significant difference between the groups was considered at $P < 0.05$.

**Supplemetary Table 4 | Gut microbiota abundance (phylum) and their correlation with clinical characteristics and biochemical variables in in the NC, HG, HF, and M cohorts.**

| | FPG | 2hPG | TG | TC | HDL | LDL |
|---|---|---|---|---|---|---|
| *Firmicutes* | 0.1051 | -0.0513 | -0.04651 | -0.1084 | 0.07876 | -0.1710 |
| *Proteobacteria* | -0.0690 | -0.1561 | 0.03583 | 0.3408* | -0.2561 | 0.3734* |
| *Bacteroidetes* | -0.0320 | 0.1740 | 0.0006 | -0.01731 | 0.001429 | 0.0558 |
| *Actinobacteria* | -0.2277 | -0.3155* | 0.1114 | 0.0734 | -0.1221 | 0.02204 |
| *Verrucomicrobia* | -0.0466 | -0.05321 | -0.03252 | 0.1199 | 0.1352 | 0.08451 |
| *Tenericutes* | -0.0234 | -0.03199 | 0.1671 | 0.1990 | -0.03193 | 0.2782 |

Data are Spearman correlation coefficients. Significant correlations shown in boldface. *P < 0.05.

**Supplementary Table 5 | P-value of significant plasma lipids of NC, HG, HF and M cohorts.**

| Compound Name | Group1 | Group2 | P-value | Compound Name | Group1 | Group2 | P-value | Compound Name | Group1 | Group2 | P-value |
|---|---|---|---|---|---|---|---|---|---|---|---|
| 3-Dehydrocarnitine | M | NC | 0.0035 | 6-Hexanoyl-D-erythro-sphingosine | M | HF | 0.0000 | 6-Hexanoyl-D-erythro-sphingosine | M | HG | 0.0003 |
| 6-Hexanoyl-D-erythro-sphingosine | M | NC | 0.0001 | ArachidoylEthanolamide | M | HF | 0.0002 | ArachidoylEthanolamide | M | HG | 0.0006 |
| ArachidoylEthanolamide | M | NC | 0.0008 | CER | M | HF | 0.0000 | CER | M | HG | 0.0001 |
| CER | M | NC | 0.0002 | cPA | M | HF | 0.0056 | DG | M | HG | 0.0005 |
| Che | M | NC | 0.0039 | DG | M | HF | 0.0000 | Eicosanoicacid | M | HG | 0.0042 |
| DG | M | NC | 0.0006 | Eicosanoicacid | M | HF | 0.0027 | FA | M | HG | 0.0048 |
| dMePE | M | NC | 0.0070 | FA | M | HF | 0.0007 | Hexadecanamide | M | HG | 0.0000 |
| Eicosanoicacid | M | NC | 0.0044 | Hexadecanamide | M | HF | 0.0000 | LdMePE | M | HG | 0.0005 |
| FA | M | NC | 0.0106 | LdMePE | M | HF | 0.0014 | LPA | M | HG | 0.0005 |
| Hexadecanamide | M | NC | 0.0000 | LPA | M | HF | 0.0001 | LPC | M | HG | 0.0243 |
| LdMePE | M | NC | 0.0005 | LPC | M | HF | 0.0471 | LPEt | M | HG | 0.0011 |
| LPA | M | NC | 0.0006 | LPEt | M | HF | 0.0113 | LPG | M | HG | 0.0027 |
| LPC | M | NC | 0.0193 | LPI | M | HF | 0.0010 | LPI | M | HG | 0.0100 |
| LPEt | M | NC | 0.0023 | MG | M | HF | 0.0016 | LPS | M | HG | 0.0067 |
| LPG | M | NC | 0.0013 | N-hexadecyl-ethanolamine | M | HF | 0.0092 | MG | M | HG | 0.0054 |
| LPI | M | NC | 0.0047 | OAHFA | M | HF | 0.0000 | N-hexadecyl-ethanolamine | M | HG | 0.0048 |
| MG | M | NC | 0.0028 | Octadecanoicacid | M | HF | 0.0042 | OAHFA | M | HG | 0.0001 |

| | | | | | | | | | | | |
|---|---|---|---|---|---|---|---|---|---|---|---|
| N-hexadecyl-ethanolamine | M | NC | 0.0018 | PAF | M | HF | 0.0055 | Octadecanoicacid | M | HG | 0.0346 |
| OAHFA | M | NC | 0.0001 | PC | M | HF | 0.0055 | PAF | M | HG | 0.0001 |
| Octadecanoicacid | M | NC | 0.0013 | PEt | M | HF | 0.0008 | PC | M | HG | 0.0008 |
| PAF | M | NC | 0.0001 | PG | M | HF | 0.0005 | PE | M | HG | 0.0202 |
| PC | M | NC | 0.0079 | PI | M | HF | 0.0002 | PEt | M | HG | 0.0001 |
| PE | M | NC | 0.0010 | PIP | M | HF | 0.0025 | PG | M | HG | 0.0002 |
| PEt | M | NC | 0.0002 | PIP3 | M | HF | 0.0093 | PI | M | HG | 0.0002 |
| PG | M | NC | 0.0000 | PS | M | HF | 0.0091 | PIP | M | HG | 0.0044 |
| phSM | M | NC | 0.0006 | So | M | HF | 0.0009 | PIP3 | M | HG | 0.0156 |
| PI | M | NC | 0.0000 | TG | M | HF | 0.0001 | PS | M | HG | 0.0034 |
| PIP | M | NC | 0.0015 | | | | | SM | M | HG | 0.0074 |
| SM | M | NC | 0.0045 | | | | | So | M | HG | 0.0021 |
| So | M | NC | 0.0007 | | | | | TG | M | HG | 0.0000 |
| TG | M | NC | 0.0000 | | | | | | | | |

Significant differences were calculated using the ANOVA test. A significant difference between the groups was considered at $P < 0.01$.

**Supplemetary Table 6 | Gut microbiota abundance (phylum) and their correlation with lipid classes variables in in the M cohort.**

| | 3-Dehydrocarnitine | CER | DG | cPA | Hexadecanamide | Octadecanoicacid | PEt | PIP3 | PC | So | SM |
|---|---|---|---|---|---|---|---|---|---|---|---|
| *Firmicutes* | 0.6247$^{*}$ | 0.6843$^{*}$ | 0.5893$^{*}$ | - | 0.6313$^{*}$ | 0.6378$^{*}$ | 0.6434$^{*}$ | 0.7909$^{**}$ | | - | - |
| *Bacteroidetes* | -0.5975$^{*}$ | -0.7636$^{**}$ | -6.3645$^{*}$ | -0.5990$^{*}$ | -0.6943$^{*}$ | | -0.7510$^{**}$ | -0.7694$^{**}$ | -0.5853$^{*}$ | | |
| *Actinobacteria* | - | - | - | - | - | - | - | - | - | -0.5899$^{*}$ | |
| *Tenericutes* | - | - | - | - | - | - | - | - | - | - | -0.6222$^{*}$ |

Data are Spearman correlation coefficients. Significant correlations shown in boldface. *P < 0.05; **P < 0.01.
